# A Case Series on the Efficacy and Safety of Transperineal Laser Ablation for Benign Prostatic Hyperplasia

**DOI:** 10.3390/jcm15020540

**Published:** 2026-01-09

**Authors:** Malone R. Locke, Donald Russell Locke

**Affiliations:** 1 Department of Urology, University of Arizona, Tucson, AZ 85724, USA; 2 Columbia Campus, University of South Carolina School of Medicine, Columbia, SC 29209, USA; 3 Vantage Urologic Institute, Ocala, FL 34481, USA

**Keywords:** transperineal laser ablation, benign prostatic hyperplasia, lower urinary tract symptoms

## Abstract

**Background/Objectives**: Traditional surgeries for benign prostatic hyperplasia (BPH), such as transurethral resection of the prostate (TURP), carry risks including sexual dysfunction and extended recovery. EchoLaser transperineal laser ablation (TPLA) offers a minimally invasive alternative with potential benefits in preserving sexual function and reducing recovery time. This exploratory study evaluated the safety and efficacy of EchoLaser TPLA for the treatment of prostate-related voiding symptoms. **Methods**: This retrospective, single-center study enrolled seven patients with lower urinary tract symptoms due to BPH. TPLA was performed under local anesthesia, and follow-up was conducted at 1, 3, 6, and 12 months. The primary outcome was measured by the International Prostate Symptom Score (IPSS). Secondary outcomes included PSA levels, prostate and transition zone (TZ) volume, Q_max_, post-void residual (PVR) volume, quality of life (QoL) score, Sexual Health Inventory for Men (SHIM) score, and Male Sexual Health Questionnaire to assess for ejaculatory dysfunction (MSHQ-EjD) score. **Results**: Statistically significant improvements in IPSS, Q_max_, PVR, and QoL relative to baseline were observed at 1 month post-treatment, and these improvements remained significant throughout the 12-month follow-up period. Post-treatment reductions in PV and TZ volume were statistically significant at 6 months; while PV was further reduced at 12 months, this change lacked statistical significance. No statistically significant post-treatment changes were observed in SHIM, MSHQ-EjD 3-Item or Bother scores, or PSA. Mean pain score on the 10-point visual analog scale for procedural pain was 2.14 ± 0.69. **Conclusions**: Although limited by a lack of generalizability, our findings are consistent with previous studies that have shown EchoLaser TPLA to be a safe and effective in-office treatment for prostate-related voiding symptoms, with minimal discomfort and negligible impact on sexual function. Further studies with larger cohorts and extended follow-up are needed.

## 1. Introduction

Benign prostatic hyperplasia (BPH) is a common condition that affects a significant portion of aging men [1]. In a recent meta-analysis, pooled prevalences of 14.8%, 20.0%, 29.1%, 36.8%, and 38.4% were observed for age groups of 40–49 years, 50–59 years, 60–69 years, 70–79 years, and 80 years and above, respectively [2]. Based on data from the 2021 global disease burden study, the number of global prevalent cases has increased by 122% since 1990, with an increased absolute burden of BPH that is driven primarily by an upward trend for BPH in low and low-middle sociodemographic regions [3].

Characterized by a nodular enlargement of the prostate due to hyperplasia of epithelial and/or stromal components [4], BPH usually affects the prostate transition zone and leads to lower urinary tract symptoms (LUTS) such as urinary frequency, nocturia, and urgency [2]. While the precise pathophysiology of BPH remains to be elucidated, relevant contributors include hormonal factors (androgens and estrogens) and genetic factors that include genes for growth factors, androgen regulation, apoptosis, and androgen-regulated genes [4]. Recent genomic characterization of BPH has discovered potentially new signaling molecules elevated in BPH tissue, including bone morphogenetic protein 5 (BMP5) and CXC chemokine ligand 13 (CXCL13), and a stromal transcriptional signature that correlates with BPH symptoms. This work also uncovered significant changes in the cell types residing in both the BPH epithelium and BPH stroma, suggesting that BPH reflects a fundamental relandscaping of cells and tissue [5]. BPH is a non-premalignant condition, in contrast to prostate cancer, in which the most common molecular alterations are *MYC* overexpression, shortening of telomeres, inactivation of the glutathione S-transferase pi1 gene (*GSTP1*) and other genes by CpG island hypermethylation, and gene fusions involving ETS transcription factors (*TMPRSS2::ERG)* [4].

Diagnostic evaluation in men with lower urinary tract symptoms (LUTS) of suspected BPH origin should include a medical history, physical examination, use of the International Prostate Symptom Score (IPSS), and urinalysis, with measurement of post-void residual and uroflowmetry if available [6]. Traditional treatments, such as transurethral resection of the prostate (TURP), are effective but come with inherent risks, including sexual dysfunction and lengthy recovery periods [7,8].

In response to these drawbacks, newer, minimally invasive treatments such as EchoLaser transperineal laser ablation (TPLA) have been introduced. EchoLaser TPLA utilizes laser energy to reduce prostate volume and improve LUTS due to BPH [9,10], while also offering the potential for preserving sexual function [11,12]. EchoLaser TPLA has shown promise due to its precision, ability to be performed under local anesthesia, and outpatient nature. Furthermore, with minimal modification, EchoLaser TPLA has shown promising results when used as focal therapy for prostate cancer [13]. While established alternatives such as robotic waterjet treatment (Aquablation), convective water vapor thermal therapy (Rezūm), and prostatic urethral lift (UroLift) have demonstrated favorable outcomes [14,15,16,17], limitations such as prostate size constraints, durability concerns, and postoperative irritative symptoms have prompted exploration of newer technologies like EchoLaser TPLA.

This exploratory study aims to evaluate the efficacy, safety, and functional outcomes of EchoLaser TPLA in patients with symptomatic BPH and compare these findings with the existing literature on similar minimally invasive treatments. Our study provides evidence that EchoLaser TPLA is a safe and effective in-office treatment for prostate-related voiding symptoms, with minimal discomfort and negligible impact on sexual function.

## 2. Materials and Methods

### 2.1. Patient Selection

This retrospective, single-center study enrolled seven patients with LUTS due to BPH. Patient accrual was continuous with no formal screening pipeline. Inclusion criteria were age > 50 years, prostate volume (PV) > 30 mL, IPSS > 12, and Q_max_ < 15 mL/s. Patients generally had symptomatic, large-volume BPH, had either failed or had suboptimal response to oral medical therapy, or preferred to avoid more invasive interventions. Exclusion criteria were previous prostate surgery, prostate cancer, active urinary tract infection, or neurogenic bladder dysfunction. The 50-year-old age limit was adopted as younger men with LUTS often have alternative etiologies or confounders that are less consistent with age-related BPH progression. This study was conducted in accordance with the principles of the Declaration of Helsinki. Written informed consent was obtained from all patients. This study was deemed to be exempt from IRB approval because it was a retrospective review of established clinical data.

### 2.2. Procedure

EchoLaser TPLA was performed using the EchoLaser laser device and Echolaser Smart Interface (ESI) tool (software version EI SW5 0005 004) (Elesta S.p.A., Calenzano, Florence, Italy). A BK Medical bkSpecto ultrasound system with an endocavity biplane transducer (BK Medical, Burlington, MA, USA) was used for real-time imaging guidance during the procedure.

Patients were placed in the lithotomy position, and a 16 Fr Foley catheter was inserted. Local anesthesia was achieved using 1% lidocaine. Preoperative gentamicin (80 mg IM) was administered. Baseline prostate dimensions, including width, height, and length, were measured to calculate the preoperative prostate volume and transition zone (TZ) volume.

The laser fibers (Fiber Optic for PLA, Elesta S.p.A., Calenzano, Florence, Italy) were inserted transperineally through 21G introducer needles (Introducer, Elesta S.p.A., Calenzano, Florence, Italy) into the right and left lobes of the prostate under ultrasound guidance. The number of fibers, pull-back technique, power settings (ranging from 3 to 5 W), and energy deployed (ranging from 1800 to 6000 J per lobe) were determined based on individual patient characteristics. Depending on the prostate shape, volume, and planned targeted ablation regions, one or more laser fibers were positioned in parallel at 8 to 10 mm apart. Distances over 10 mm from the urethral wall, 15 mm from the bladder neck, and 10 mm from the prostatic capsule outer edge were maintained. The simultaneous use of multiple fibers enabled an increased ablation volume and allowed both prostatic lobes to be treated concurrently. A 10-point visual analog scale (VAS) was used to assess patient-reported pain during the procedure. Patients were observed for complications and were discharged the same day.

### 2.3. Follow-Up and Outcome Measures

Patients were followed up at 1, 3, 6, and 12 months post-treatment. The primary outcome measure, defined a priori, was IPSS at 12 months [18]. Secondary outcomes included prostate-specific antigen (PSA) levels, prostate volume (PV) and TZ volume, maximum flow rate (Q_max_), post-void residual (PVR) volume, quality of life (QoL) score, Sexual Health Index for Men (SHIM) score [19], and Male Sexual Health Questionnaire-Ejaculatory Dysfunction (MSHQ-EjD) score [20]. Measurements of PSA, PV, and TZ volume were not performed at 1 month and 3 months because these parameters are not routinely measured at these timepoints outside of research protocols.

### 2.4. Statistical Analysis

Data were expressed as means and standard deviations (SDs) or as medians and 95% confidence intervals (CIs). Comparisons between baseline and follow-up data were performed using Wilcoxon signed-rank tests for paired, non-normally distributed data with small sample sizes. Matched rank-biserial effect sizes were also calculated. A *p*-value <0.05 was considered statistically significant. All statistical analyses were performed using Microsoft^®^ Excel for Mac version 16.97 (Microsoft, Redmond, WA, USA).

## 3. Results

The patient cohort consisted of seven males with a mean ± SD age of 78.3 ± 10.5 years. The mean body mass index (BMI) was 25.6 ± 1.4 kg/m^2^, median (95% CI) PV was 105.80 (34.80–114.00), and median (95% CI) preoperative PSA level was 3.65 (2.00–5.90) ng/mL. One patient had an indwelling catheter at the time of the procedure, due to chronic urinary retention. One patient was receiving anticoagulant therapy. Regarding preoperative BPH medical therapy, four patients were being treated with alpha-blockers, and three patients were receiving combination therapy (alpha-blocker and 5⍺-reductase inhibitor).

The total energy deployed per patient ranged from 3600 to 12,000 J, with a mean of 9600 ± 3301.9 J. The procedure time varied between 30 and 50 min, with a mean of 42.9 ± 8.1 min. The mean VAS pain score post-TPLA procedure was 2.14 ± 0.69.

**Figure 1:**
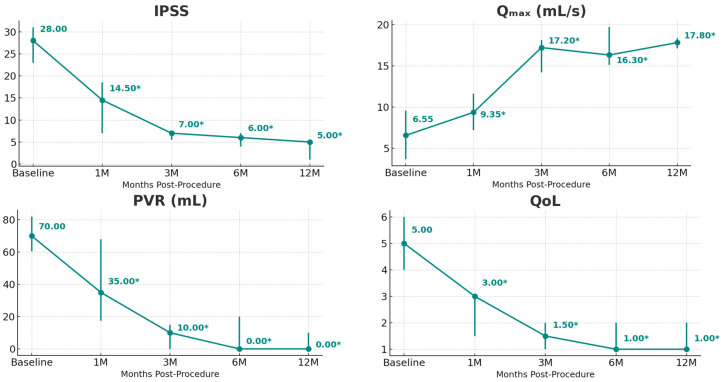
Clinical outcomes. International Prostate Symptom Score (IPSS), maximum urinary flow rate (Q_max_), post-void residual (PVR), and quality of life (QoL). Statistically significant values (*p* < 0.05) are denoted with an asterisk (*).

**Figure 2:**
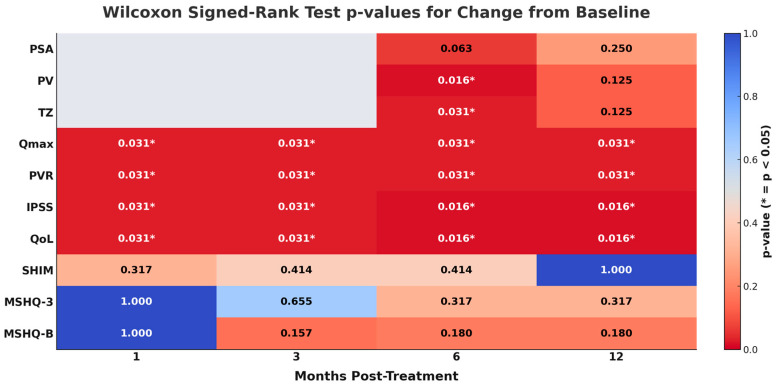
Heatmap of *p*-values for each variable compared vs. baseline across timepoints.

All patients discontinued BPH medical therapy, and at 12-month follow-up, none had required resumption of this medication. Improvements in IPSS, Q_max_, PVR, and QoL relative to baseline were statistically significant at 1 month post-treatment and remained significant at all timepoints during the 12-month follow-up period (Table 1 and Figure 1 and Figure 2). For these parameters, the 12-month changes vs. baseline were as follows: IPSS, 5.00 vs. 28.00 (*p* = 0.016); Q_max_, 17.8 vs. 6.55 mL/s (*p* = 0.031); PVR, 0 vs. 70.0 mL (*p* = 0.031); and QoL, 1.00 vs. 5.00 (*p* = 0.016). For IPSS, Q_max_, and PVR, the majority of the treatment response was observed at 3 months post-treatment, although incremental benefit was observed at 12 months. For QoL, the improvement observed at 3 months plateaued beyond this timepoint (Figure 1). PV and TZ volume were statistically significant vs. baseline at 6 months, but not at 12 months. Throughout the follow-up period, PSA levels and SHIM and MSHQ scores were not statistically different from baseline.

One patient experienced complications necessitating clean intermittent catheterization (CIC) due to failed voiding trials at 1 month post-treatment. The same patient also developed a urinary tract infection that was managed with ciprofloxacin. Other reported adverse events at 1 month included moderate dysuria (*n* = 1), bladder spasms (*n* = 1), moderate urinary frequency and urgency treated with oxybutynin (*n* = 1), and mild urinary frequency and urgency (*n* = 1). No adverse events were reported at 3, 6, or 12 months.

## 4. Discussion

In this exploratory study, we evaluated the efficacy and safety of EchoLaser TPLA in the management of LUTS associated with BPH in a cohort of seven patients treated in routine practice. Our results demonstrate significant improvements in urinary symptoms and QoL and preservation of sexual function following EchoLaser TPLA treatment, with the most notable changes occurring by 3 months post-procedure and persisting through 12 months.

Patients treated with EchoLaser TPLA exhibited marked improvements in Q_max_, PVR, IPSS, and QoL scores. These clinical endpoints, central to the management of BPH, showed rapid and statistically significant changes that were maintained throughout the study period. Q_max_ improved from a baseline median of 6.55 mL/s to 17.20 mL/s at 3 months, indicating a dramatic enhancement in urinary flow. This trend continued through 12 months, where median Q_max_ reached 17.80 mL/s, representing nearly a threefold improvement from baseline. PVR followed a similar trajectory, declining from a median of 70.0 mL at baseline to 10.00 mL at 3 months and further decreasing to 0 mL by 12 months, reflecting near-complete bladder emptying and suggesting meaningful relief of obstruction. Of note, median PV at 12 months was numerically smaller than at 3 months; however, the 12-month value lacked the statistical significance that was observed at 3 months. A similar observation has previously been reported by Laganà et al. in a prospective single-center experience of EchoLaser TPLA in a cohort of 63 patients [21]. Possible explanations for this lack of statistical significance at 12 months include measurement variability or transient tissue changes.

Symptom burden, as measured by the IPSS, demonstrated a steep reduction from a baseline median of 28.00 to 7.00 at 3 months and 5.00 at 12 months. These values fall into the range of mild symptoms, indicating a substantial and sustained improvement in patient-reported outcomes. Consistent with this observation, no patient required resumption of their BPH medication during the 12 months of follow-up. Parallel improvements were observed in QoL scores, which dropped from a baseline median of 5.0 to 1.50 by 3 months and remained low at 1.00 at 12 months. This alignment between objective voiding metrics and subjective symptom relief supports the overall effectiveness of EchoLaser TPLA.

Importantly, these changes occurred rapidly—often by 1 month—and remained stable or improved over time, suggesting a durable therapeutic effect without evidence of symptom rebound. This may distinguish EchoLaser TPLA from other minimally invasive treatments such as convective water vapor thermal therapy or prostatic urethral lift, which may take longer to reach maximal effect [22] or be limited by prostate size [7,8] or irritative voiding symptoms [11,16,22] in the early post-procedural period.

The incidence of complications was low in this cohort (one case each of failed voiding, moderate dysuria, bladder spasms, moderate urinary frequency and urgency, and mild urinary frequency and urgency). Importantly, no adverse events were reported at 3, 6, or 12 months, supporting the favorable safety profile of the procedure over time. These data are consistent with the overall ‘benign’ safety profile of EchoLaser TPLA that has emerged from the increasing number of studies of this minimally invasive procedure [10,23]. Safety advantages of EchoLaser TPLA include the technique’s transperineal access which avoids the need for urethral instrumentation and associated risk, the ability of the operator to be aided by preprocedural simulation software, and real-time visualization of the thermoblation, which facilitates preservation of key anatomical structures [24,25].

The limitations of this study include its small sample size and single-arm design, which may affect the generalizability of the results and preclude direct comparisons with alternative therapies. However, recent randomized controlled trials have reported direct comparisons of EchoLaser TPLA vs. TURP and separately vs. convective water vapor thermal therapy. In one trial, which used changes in ejaculatory function as the primary endpoint, EchoLaser TPLA was associated with a significantly greater preservation of ejaculatory function compared with TURP [11], and in a second trial, the MSHQ–ejaculatory function domain scores of patients who underwent EchoLaser TPLA were not significantly worse at 12-month follow-up, in contrast to the significant worsening observed for TURP patients [12]. In both trials, both EchoLaser TPLA and TURP produced significant improvements in measures of urinary symptoms and urinary function; however, post-treatment improvements in Q_max_ were greater for TURP than for EchoLaser TPLA. In a randomized comparison of EchoLaser TPLA vs. convective water vapor thermal therapy, the two techniques produced comparable improvements in functional outcomes, with EchoLaser TPLA appearing to produce a faster improvement in patient-reported outcomes [22]. Moreover, in the subset of sexually active patients in this trial, both techniques preserved erectile function and improved MSHQ-EjD scores at 6-month follow-up [26]. In addition, the follow-up period, while encouraging in demonstrating sustained benefit at one year, does not establish long-term durability beyond 12 months in our cohort. However, a recently published study of 40 men with symptomatic BPH who underwent EchoLaser TPLA provided evidence of durable benefits across a range of clinical outcomes during 56.5 months median duration follow-up [27]. Another study demonstrated the benefits of EchoLaser TPLA at 3-year follow-up in 20 patients [28]. Despite these limitations, our findings provide early evidence that EchoLaser TPLA offers rapid, durable symptom relief and QoL improvement with a favorable safety profile. These results support continued evaluation of EchoLaser TPLA in larger, controlled studies.

## 5. Conclusions

Our study findings have limited generalizability due to the small sample size and single-center, retrospective design. Nevertheless, our results are consistent with those of previous studies, which have shown EchoLaser TPLA to be a safe, effective, and minimally invasive treatment for patients with BPH. EchoLaser TPLA led to significant improvements in objective measures such as Q_max_ and PVR, as well as sustained reductions in IPSS and QoL scores over 12 months. Adverse events were minimal and resolved early in the follow-up period, and no patients required retreatment.

These outcomes suggest that EchoLaser TPLA may offer a durable therapeutic benefit with a favorable safety profile, making it an attractive alternative to traditional surgical options like TURP and to other minimally invasive therapies, particularly in patients with larger prostates or a desire to avoid prolonged catheterization or sexual side effects.

Further research with larger cohorts and extended follow-up is necessary to confirm these findings and to directly compare EchoLaser TPLA with other BPH treatment modalities. Nonetheless, TPLA represents a promising addition to the armamentarium of BPH management, offering meaningful symptom relief with low morbidity and rapid recovery.

## Figures and Tables

**Table 1:** Median values and 95% confidence intervals for each variable at baseline and at 1, 3, 6, and 12 months for patients with recorded data at each timepoint. Matched rank-biserial effect sizes are also reported.

Variable	Timepoint				
	Baseline	1 Month	3 Months	6 Months	12 Months
PSA (ng/mL)	3.65 (2.00–5.90)	ND	ND	2.85 (0.97–3.50) r = −0.90	1.87 (0.90–3.80) r = −0.80
PV (mL)	105.80 (34.80–114.00)	ND	ND	76.93 (25.00–82.13) * r= −1.00	55.34 (49.88–62.94) r = −1.00
TZ volume (mL)	65.35 (21.30–68.02)	ND	ND	54.19 (19.60–57.41) * r= −0.93	41.02 (35.27–43.41) r = −1.00
Q_max_ (mL/s)	6.55 (3.65–9.55)	9.35 (7.20–11.60) * r = 1.00	17.20 (14.20–18.10) * r = 1.00	16.30 (15.10–19.70) * r = 1.00	17.80 (17.10–18.30) * r = 1.00
PVR (mL)	70.00 (60.50–82.00)	35.00 (17.50–68.00) * r = −1.00	10.00 (0.00–15.00) * r = −1.00	0.00 (0.00–20.00) * r = −1.00	0.00 (0.00–10.00)* r = −1.00
IPSS	28.00 (23.00–31.00)	14.50 (7.00–18.50) * r = −1.00	7.00 (5.50–7.50) * r = −1.00	6.00 (4.00–7.00) * r = −1.00	5.00 (1.00–5.00)* r = −1.00
QoL	5.00 (4.00–6.00)	3.00 (1.50–3.00) * r = −1.00	1.50 (1.00–2.00) * r = −1.00	1.00 (1.00–2.00) * r = −1.00	1.00 (1.00–2.00)* r = −1.00
SHIM	1.00 (1.00–16.00)	1.00 (1.00–15.00) r = −1.00	1.00 (1.00–15.00) r = −0.50	1.00 (1.00–14.00) r = −0.50	1.00 (1.00–15.00) r = 0.00
MSHQ-3	1.00 (1.00–4.00)	1.00 (1.00–4.00) r = 0.00	1.00 (1.00–4.00) r = 0.33	1.00 (1.00–6.00) r = 1.00	1.00 (1.00–6.00) r = 1.00
MSHQ-B	1.00 (1.00–3.00)	1.00 (1.00–3.00) r = 0.00	1.00 (1.00–2.00) r = −1.00	1.00 (1.00–2.00) r = −1.00	1.00 (1.00–2.00) R =−1.00

Statistically significant values (*p* < 0.05) are denoted with an asterisk (*). Abbreviations: ND, not determined; PSA, prostate-specific antigen; PV, prostate volume; TZ, transition zone; Q_max_, maximum urinary flow rate; PVR, post-void residual; IPSS, International Prostate Symptom Score; QoL, quality of life; SHIM, Sexual Health Inventory for Men questionnaire; MSHQ, Male Sexual Health Questionnaire for Ejaculatory Dysfunction (3-Item and Bother scores).

## Data Availability

The data presented in this study are available on request from the corresponding author due to protected health information in patient medical records.

## Abbreviations

The following abbreviations are used in this manuscript:

BPH	Benign prostatic hyperplasia
IPSS	International Prostate Symptom Score
LUTS	Lower urinary tract symptoms
MSHQ-EjD	Male Sexual Health Questionnaire-Ejaculatory Dysfunction
PSA	Prostate-specific antigen
PV	Prostate volume
PVR	Post-void residual
QoL	Quality of life
SD	Standard deviation
SHIM	Sexual health in men
TPLA	Transperineal laser ablation
TURP	Transurethral resection of the prostate
TZ	Transition zone
